# Genetically predicted immune cells mediate the association between gut microbiota and neuropathy pain

**DOI:** 10.1007/s10787-024-01514-y

**Published:** 2024-07-02

**Authors:** Zhixuan Lan, Yi Wei, Kan Yue, Ruilin He, Zongbin Jiang

**Affiliations:** Department of Pain Medicine, The Second Affiliated Hospital of Guangxi Medical University, Guangxi, Nanning, 530005 China

**Keywords:** Gut microbiota, Neuropathic pain, Immune cells, Causal relationship, Mendelian randomization study

## Abstract

**Background:**

Previous observational studies have indicated a complex association between gut microbiota (GM) and neuropathic pain (NP). Nonetheless, the precise biological mechanisms underlying this association remain unclear. Therefore, we adopted a Mendelian randomization (MR) approach to investigate the causal relationship between GM and neuropathic pain including post-herpetic neuralgia (PHN), painful diabetic peripheral neuropathy (PDPN), and trigeminal neuralgia (TN), as well as to explore the potential mediation effects of immune cells.

**Methods:**

We performed a two-step, two-sample Mendelian randomization study with an inverse variance-weighted (IVW) approach to investigate the causal role of GM on three major kinds of NP and the mediation effect of immune cells between the association of GM and NP. In addition, we determine the strongest causal associations using Bayesian weighted Mendelian randomization (BWMR) analysis. Furthermore, we will investigate the mediating role of immune cells through a two-step Mendelian randomization design.

**Results:**

We identified 53 taxonomies and pathways of gut microbiota that had significant causal associations with NP. In addition, we also discovered 120 immune cells that exhibited significant causal associations with NP. According to the BWMR and two-step Mendelian randomization analysis, we identified the following results CD4 on CM CD4 + (maturation stages of T cell) mediated 6.7% of the risk reduction for PHN through the pathway of fucose degradation (FUCCAT.PWY). CD28 + DN (CD4-CD8-) AC (Treg) mediated 12.5% of the risk reduction for PHN through the influence on *Roseburia inulinivorans*. CD45 on lymphocyte (Myeloid cell) mediated 11.9% of the risk increase for TN through the superpathway of acetyl-CoA biosynthesis (PWY.5173). HLA DR + CD8br %T cell (TBNK) mediated 3.2% of the risk reduction for TN through the superpathway of GDP-mannose-derived O-antigen building blocks biosynthesis (PWY.7323). IgD-CD38-AC (B cell) mediated 7.5% of the risk reduction for DPN through the pathway of thiazole biosynthesis I in E. coli (PWY.6892).

**Discussion:**

These findings provided evidence supporting the causal effect of GM with NP, with immune cells playing a mediating role. These findings may inform prevention strategies and interventions directed toward NP. Future studies should explore other plausible biological mechanisms.

## Introduction

According to International Association for the Study of Pain (IASP), neuropathic pain (NP) arises from injury or ailment affecting the somatosensory system (Jensen et al. 2011). NP is categorized into peripherally induced neuropathic pain (pNP) and central neuropathic pain (Dworkin et al. 2003; Colloca et al. 2017), with pNP being more prevalent and typically resulting from peripheral nerve damage. Conditions such as painful diabetic peripheral neuropathy (PDPN), trigeminal neuralgia (TN) and post-herpetic neuralgia (PHN) are major causes of pNP (Bril et al. 2011, 2022, 2021; Yang et al. 2019b; Qing-jun., 2018; Consensus Workgroup on Herpes Zoster 2022). NP not only causes considerable suffering for patients but also places a significant economic burden. Current treatment strategies, which often rely on widespread opioid usage, often fail to provide sufficient pain relief (Schaefer et al. 2014; Yu et al. 2019; Attal et al. 2023).

In recent studies, there has been a growing emphasis on the complex interplay between the gut microbiome (GM) and neurological conditions, particularly its involvement in the onset and regulation of NP (Gonzalez-Alvarez et al. 2023; Li et al. 2023; Moloney et al. 2016). Evidence suggests a tight connection between the GM composition and NP, with gut bacteria modulating pain sense through immunomodulation, inflammatory response regulation, and neural pathway activation (Defaye et al. 2020; Guo et al. 2019; Min 2023; Ustianowska et al. 2022). The microbiota–gut–brain axis underscores a two-way communication system, involving immune, neural, endocrine, and metabolic signaling pathways, which play a significant role in disease advancement, particularly in NP (Lin et al. 2020; Nagamine 2023; Magni et al. 2023; Moloney et al. 2016). Despite the importance of these discoveries, precise mechanisms by which gut microbiota interact with neural and immune components in NP remain elusive, with existing studies indicating correlation rather than causation (Gonzalez-Alvarez et al. 2023; Li et al. 2023). Moreover, the diversity in GM investigations and the impact of confounding variables present hurdles for clinical implementation (Lin et al. 2020; Ustianowska et al. 2022; Huang et al. 2019; Yang et al. 2019a). Recent studies also emphasize the crucial involvement of immune cells in NP, with activation of microglia, mast cells, as well as participation of immune responses in early stage inflammation, being associated with NP severity (Rahman-Enyart et al. 2022; Inoue and Tsuda 2018; Barcelon et al. 2019; Colloca et al. 2017; Magni et al. 2023; Bethea and Fischer 2021; Thacker et al. 2007). The modulation of immune cells by gut microbiota, notably through short-chain fatty acids (SCFAs) and pathogen-associated molecular patterns (PAMPs), implies a central involvement in the initiation of NP (Dworsky-Fried et al. 2020; Magni et al. 2023; Barcelon et al. 2019; Lin et al. 2020; Ustianowska et al. 2022; Ramakrishna et al. 2019; Reichenberger et al. 2013; Ding et al. 2021; Fiore et al. 2023).However, intricate interplay underlying GM, immune cells, and NP, compounded by potential confounders, calls for further exploration (Lin et al. 2020; Sun et al. 2023).

Mendelian randomization (MR) leverages genetic variants to emulate the framework of randomized controlled trials, offering strong method for delineating causal relationships between diseases and potential risk factors (Liang and Fan 2023; Skrivankova et al. 2021). The accessibility of Genome-Wide Association Studies (GWAS) data has greatly bolstered the utilization of MR in probing intricate diseases, presenting unparalleled opportunities to examine the causal relationships between GM, immune cells, and NP (Wang et al. 2024a; Lopera-Maya et al. 2022). While progress has been made in comprehending the link between gut microbiota and NP, the precise causal effects mediated by immune cells have yet to be thoroughly investigated (Min 2023; Inoue and Tsuda 2018; Guo et al. 2023; Ding et al. 2021; Chen and Tang 2023). This study employs two-sample Mendelian randomization (TSMR) and Bayesian weighted Mendelian randomization (BWMR) to explore the underlying causal relationships among them. By employing these methods, our objective was to elucidate the intermediary function of immune cells in the correlation between GM and NP, thus providing fresh perspectives for identifying biomarkers and advancing novel treatments for NP.

## Method

### Study design

**Fig. 1:**
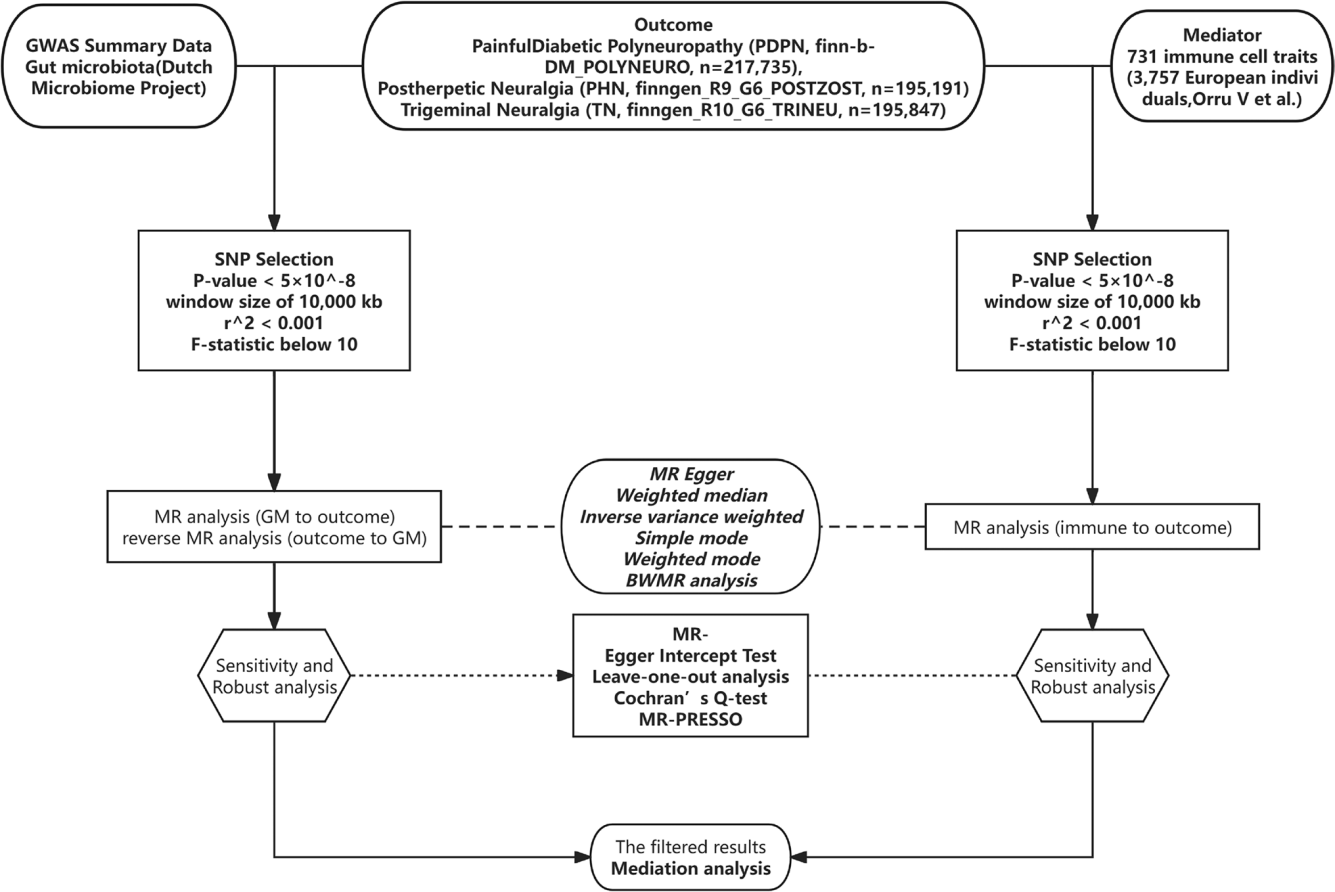
The study designs. A two-step Mendelian randomization study of GM on NP mediated by immune cell. *GM* gut microbiota, *NP* neuropathic pain, *MR* Mendelian randomization, *BWMR* Bayesian weighted Mendelian randomization, *SNP* single nucleotide polymorphism

We employed TSMR to investigate causal relationships among GM, immune cells, and three major conditions for NP: PHN, PDPN, and TN. This analysis aimed to establish the causality exerted by GM on immune cells and subsequently on these NP conditions. For supporting causal associations in TSMR, BWMR analyses were conducted to further elucidate significant causal links. (Fig. 1**).**

### GWAS summary data sources

All data utilized were sourced from online databases, with participants of GWAS being of European ancestry. Specifically, GM information was obtained from Netherlands Microbiome Project, where Esteban et al. reported on 412 gut microbial taxa. This research encompassed 7738 participants, conducting a GWAS study on 207 taxa and 205 pathways reflecting microbiome composition and activity (Lopera-Maya et al. 2022). GWAS summary data for Dutch Microbiome Project (DMP) integrated information on 5 phyla, 10 classes, 13 orders, 26 families, 48 genera, and 105 species. Further details are available in Supplementary Material Table S1.

Information regarding immune cells included 731 phenotypes, covering median fluorescence intensity (MFI, *n* = 389), absolute cell count (AC, *n* = 118), relative cell count (RC, *n* = 192), and morphological parameters (MP, *n* = 32). The first three categories covered data on various cell types, including bone marrow cells, B cells, mature T cells, monocytes, TBNK (T cells, B cells, natural killer cells), and Treg cell populations, while the last category pertained to CDC and TBNK groups. Initial GWAS analyses were centered on immune traits, utilizing samples from 3757 individuals of European ancestry, with no cohort overlap (Orrù et al. 2020). To refine the accuracy of genotype data, approximately 22 million single nucleotide polymorphisms (SNPs) were imputed using a reference panel derived from Sardinian sequences (Sidore et al. 2015). Moreover, potential confounding factors such as gender, age, and the square of age were taken into account in the evaluation of associations.

As there is no dedicated database specifically for PDPN and considering that the majority of cases within the diabetic peripheral neuropathy category are typically associated with painful symptoms, we opted to utilize diabetic polyneuropathy data as a proxy for PDPN. This choice is made on the basis that the available dataset likely encompasses a significant proportion of PDPN cases, thus facilitating meaningful analysis and insights into this condition (Bril et al. 2011). Data on painful diabetic polyneuropathy (PDPN, finn-b-DM_POLYNEURO, *n* = 375,482, https://storage.googleapis.com/finngen-public-data-r9/summary_stats/finngen_R9_DM_POLYNEURO.gz), post-herpetic neuralgia (PHN, finngen_R9_G6_POSTZOST, *n* = 330,690, https://storage.googleapis.com/finngen-public-data-r9/summary_stats/finngen_R9_G6_POSTZOST.gz), and trigeminal neuralgia (TN, finngen_R10_G6_TRINEU, *n* = 362,315, https://storage.googleapis.com/finngen-public-data-r10/summary_stats/finngen_R10_G6_TRINEU.gz) were sourced from the FinnGen consortium’s GWAS summary data, available on FinnGen website (Kurki et al. 2023; Wei et al. 2023; Liang and Fan 2023). As this study relies solely on publicly available aggregated data, no further ethical approval or participant consent was required. Further details are available in Supplementary Material Table S1.

### Instrumental variable selection and data harmonization

We selected SNPs across the genome that exhibited significant associations with our traits of interest (*P* value < 1e-5) to serve as instrumental variables (IVs). These SNPs were clustered based on their linkage disequilibrium (LD) levels, using a window size of 10,000 kb and an r^2 threshold < 0.001, with LD data sourced from the 1000 Genomes Project for European samples (Abecasis et al. 2010). When exposure SNPs were absent in the dataset, proxy SNPs were used. Both palindromic and ambiguous SNPs were excluded to prevent ambiguity in the Mendelian randomization analysis. The validity of IVs was assessed using the F-statistic, calculated as the ratio of the effect size (β) to the square of its standard error. SNPs showing an F-statistic below 10, indicating weak instruments, were excluded from further analysis. (Supplementary Material Table S2, S3, and S4).

### Primary statistical analysis

Statistical analyses were performed using R software (version 4.3.2) utilizing the TwoSampleMR, MR-PRESSO, and MendelianRandomization packages (Yavorska and Burgess 2017; Hemani et al. 2018; Verbanck et al. 2018). For the estimation of causal effects, we utilized the inverse variance-weighted (IVW) method to combine Wald ratio for each SNP in a meta-analysis framework. To complement the IVW analysis, we applied various algorithms including MR-Egger, weighted median, simple mode, weighted mode, BWMR, and MR-PRESSO (Bowden et al. 2015, 2016; Verbanck et al. 2018; Zhao et al. 2020). BWMR analysis accounted for uncertainty associated with weak instruments and addressed horizontal pleiotropy estimated in GWAS (Zhao et al. 2020).

To ensure thorough filtering, we employed a range of methods, aiming to establish a causal relationship between exposure and outcome based on the presence of consistent positive results from both the IVW method and Bayesian weighted analysis (Hemani et al. 2018; Chen et al. 2020). Sensitivity analyses were conducted to evaluate horizontal pleiotropy and heterogeneity using three approaches: the MR-Egger Intercept Test, Mendelian Randomization Pleiotropy RESidual Sum and leave-one-out analysis, and Cochran’s Q test, thereby bolstering the robustness of our findings (Bowden et al. 2015; Long et al. 2023). MR-PRESSO allows for detecting overall horizontal pleiotropy among IVs and also identifies outliers causing pleiotropy (Verbanck et al. 2018). *P* values > 0.05 in both MR-Egger Intercept Test and MR-PRESSO indicated pleiotropy absence. Cochran’s Q test indicated non-heterogeneity with *P* values > 0.05. Microbial communities showing signs of horizontal pleiotropy or heterogeneity were omitted from the analysis. Leave-one-out analysis was performed to investigate the effects of potential outlier genetic variants, thereby mitigating influences from heterogeneity and horizontal pleiotropy.

### Mediation analysis

**Fig. 2:**
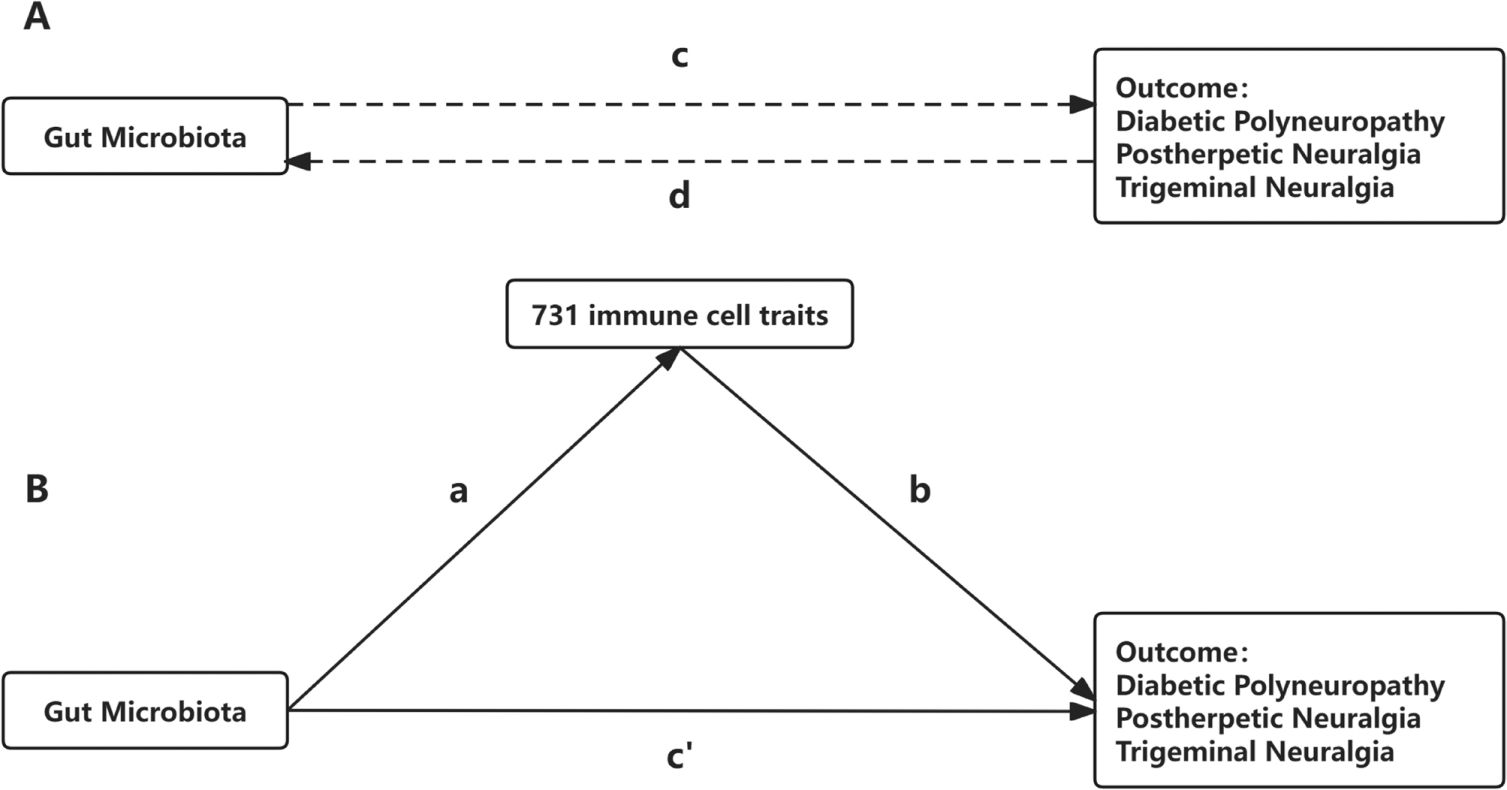
Mediation analysis. **A** The total effect between Gut microbiota (GM) and Neuropathic Pain (NP), where c is the total effect using genetically predicted GM as exposure and NP as outcome and d is the total effect using genetically predicted NP as exposure and GM as outcome. **B** The total effect was decomposed into: (i) indirect effect using a two-step approach (where a is the total effect of GM on immune cell, and b is the effect of immune cell on NP) and the product method (a × b) and (ii) direct effect (c′ = c – a × b). Proportion mediated was the indirect effect divided by the total effect

To delve deeper into the potential of immune cells within the causal pathway linking GM to NP outcomes, we implemented TSMR framework for mediation analysis. An advanced analytical approach enabled the disentanglement of the overall effect into indirect (i.e., mediated through the intermediary of immune cells) and direct (i.e., independent of the mediator) components (Carter et al. 2021), as shown in Fig. 2.

More precisely, the aggregate influence of GM on NP was dissected into (1) a direct effect of GM on NP, represented as path c in Fig. 2A, and (2) an indirect effect mediated through immune cells, denoted as a × b path in Fig. 2B. This methodology facilitated the estimation of the mediation effect as a percentage of the total effect by comparing the magnitude of the indirect effect against the total effect. Moreover, the 95% confidence intervals (CI) for the mediation effect were accurately computed using the delta method, ensuring the accuracy and robustness of our findings (Michael Lynch 2001).

## Result

### Causal effects of GM on NP

**Fig. 3:**
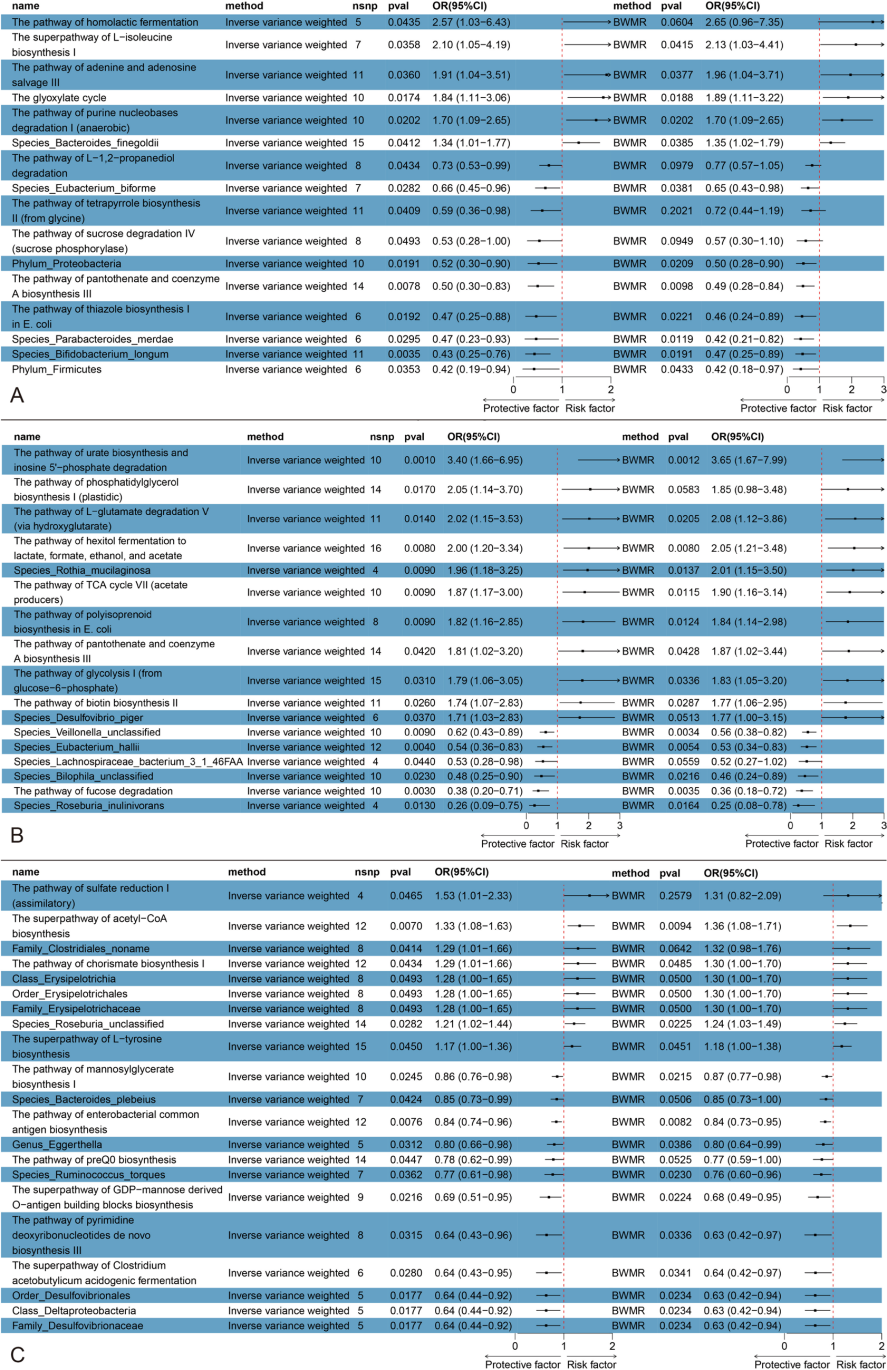
MR analysis showed the causality of GM on NP was significant. **A**: MR analysis of GM and PDPN (IVW and BWMR). **B**: MR analysis of GM and PHN (IVW and BWMR). **A**: MR analysis of GM and TN (IVW and BWMR). *CI* confidence interval, *GM* gut microbiota, *MR* Mendelian randomization, *OR* odds ratio, *nsnp* single nucleotide polymorphism, *IVW* inverse variance-weighted, *BWMR* Bayesian weighted Mendelian randomization

Significant IVW results for the taxonomic units of the gut microbiome are displayed in a forest plot. Utilizing a threshold of *P* < 1e-5, MR analysis was conducted on 412 GM taxa, identifying 53 microbial taxa and pathways associated with PHN, PDPN, or TN as identified through the IVW method. (Detailed data can be found in the supplementary materials table S5-S9 and Fig. 3).

### Causal effects of GM on PDPN

In our study, we identified six bacterial groups and metabolic pathways significantly associated with an increased risk of PDPN. The most significant were *Bacteroides finegoldii* (OR_IVW: 1.339, 95% CI: 1.012 to 1.773, P_IVW = 0.041, P_BW = 0.039) and the glyoxylate bypass pathway (PWY_GLYOXYLATE.BYPASS) (OR_IVW: 1.845, 95% CI: 1.114 to 3.056, P_IVW = 0.017, P_BW = 0.019). In addition, we found ten bacterial groups and metabolic pathways significantly associated with a decreased risk of PDPN. The most notable among these were *Bifidobacterium longum* (OR_IVW: 0.433, 95% CI: 0.246 to 0.759, P_IVW = 0.004, P_BW = 0.019) and the biosynthesis pathway of pantothenate and coenzyme A III (PWY.4242) (OR_IVW: 0.498, 95% CI: 0.298 to 0.832, P_IVW = 0.008, P_BW = 0.010). (Fig. 3A).

### Causal effects of GM on PHN

In our MR analysis of GM communities associated with PHN, we identified 11 bacterial groups and metabolic pathways significantly associated with an increased risk of PHN. *Rothia mucilaginosa* from the *Rothia* genus (OR_IVW: 1.960, 95% CI: 1.181 to 3.252, P_IVW = 0.009, P_BW = 0.014) was found to have the most significant association with increased PHN risk. In terms of metabolic pathways, the biosynthesis of uric acid, particularly through the degradation of inosine 5'-phosphate (PWY.5695), (OR_IVW: 3.398, 95% CI: 1.661 to 6.950, P_IVW = 0.001, P_BW = 0.001), showed the strongest association with increased risk of PHN. However, we also identified six bacterial groups and metabolic pathways significantly related to a decreased risk of PHN. The most notable among these were *Eubacterium hallii* (OR_IVW: 0.541, 95% CI: 0.355 to 0.826, P_IVW = 0.004, P_BW = 0.005) and the degradation process of fucoidan (FUCCAT.PWY) (OR_IVW: 0.381, 95% CI: 0.203 to 0.714, P_IVW = 0.003, P_BW = 0.003). (Fig. 3B).

### Causal effects of GM on TN

In our MR analysis of gut microbial communities associated with TN, we identified nine bacterial groups and metabolic pathways significantly associated with an increased risk of TN. The most significant among these were an unclassified strain of the *Roseburia* genus (OR_IVW: 1.213, 95% CI: 1.021 to 1.440, P_IVW = 0.028, P_BW = 0.022) and the superpathway of acetyl-CoA biosynthesis (PWY.5173) (OR_IVW: 1.328, 95% CI: 1.080 to 1.631, P_IVW = 0.007, P_BW = 0.009). We also identified 12 bacterial groups and metabolic pathways significantly associated with a decreased risk of TN. Among these, the most notable were bacteria from the order Desulfovibrionales (OR_IVW: 0.637, 95% CI: 0.439 to 0.925, P_IVW = 0.018, P_BW = 0.023) and the biosynthetic pathway for the common antigen in Enterobacteriaceae (ECA) (ECASYN.PWY) (OR_IVW: 0.844, 95% CI: 0.745 to 0.956, P_IVW = 0.008, P_BW = 0.008 (Fig. 3C).

### Causal effects of immune cells on PDPN

**Fig. 4:**
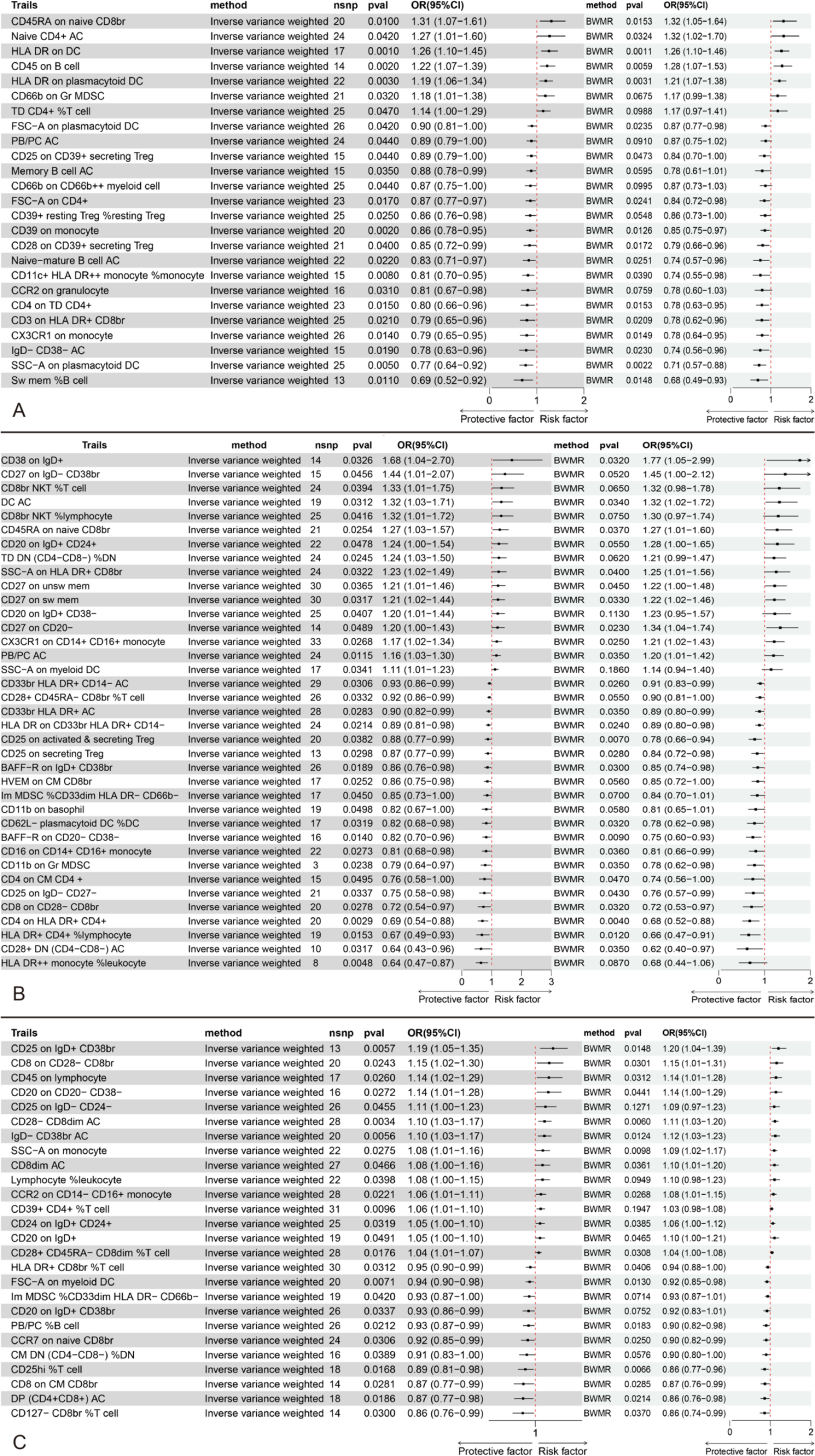
MR analysis showed the causality of immune cell on NP was significant. **A**: MR analysis of immune cell and PDPN (IVW and BWMR). **B**: MR analysis of immune cell and PHN (IVW and BWMR). **A**: MR analysis of immune cell and TN (IVW and BWMR). *CI* confidence interval, *MR* Mendelian randomization, *OR* odds ratio, *nsnp* single nucleotide polymorphism, *IVW* inverse variance-weighted, *BWMR* Bayesian weighted Mendelian randomization

In our MR analysis of immune cells associated with PDPN, we identified 27 immune cells significantly associated with an increased risk of PDPN. These included five types in the maturation stages of T cell group, three in the Myeloid cell group, three in the TBNK group, three in the B cell group, five in the cDC group, five in the Treg group, and three in the Monocyte group. The strongest significant association was noted with HLA DR on DC (from the cDC panel) (OR_IVW: 1.468, 95% CI: 1.271 to 1.696, P_IVW = 1.77E-07, P_BW = 0.006). In addition, we identified 30 immune cells associated with a decreased risk of PDPN, including 8 in the B cell group, 6 in the cDC group, 5 in the Treg group, 4 in the TBNK group, 3 in the Myeloid cell group, and 3 in the Maturation stages of T cell group. The most significant association was with CD39 on monocyte (from the Treg panel) (OR_IVW: 0.848, 95% CI: 0.756 to 0.951, P_IVW = 0.012, P_BW = 0.013) (Fig. 4A).

### Causal effects of immune cells on PHN

In our MR analysis of immune cells associated with PHN, we identified 16 immune cells significantly associated with an increased risk of PHN. These include eight in the B cell group, three in the TBNK group, two in the Maturation stages of T cell group, two in the cDC group, and one in the Monocyte group. Notably, PB/PC AC (B cell panel) exhibited the strongest significant association (OR_IVW: 1.160, 95% CI: 1.034 to 1.301, P_IVW = 0.012, P_BW = 0.035). In addition, we identified 21 immune cells related to a decreased risk of PHN, with 6 in the myeloid cell group, 3 in the TBNK group, 5 in the Treg group, 3 in the B cell group, 2 in the Maturation stages of T cell group, 1 in the Monocyte group, and 1 in the cDC group. Among these, the most significant was CD4 on HLA DR + CD4 + (TBNK panel) (OR_IVW: 0.689, 95% CI: 0.539 to 0.881, P_IVW = 0.003, P_BW = 0.004) (Fig. 4B).

### Causal effects of immune cells on TN

In our MR analysis of immune cells related to TN, we identified 15 immune cells significantly associated with an increased risk of TN. These included six in the B cell group, four in the Treg group, two in the TBNK group, and one each in the Monocyte, Myeloid cell, and cDC groups. The strongest significant association was observed with CD28- CD8dim AC (Treg panel) (OR_IVW: 1.099, 95% CI: 1.032 to 1.172, P_IVW = 0.003, P_BW = 0.006). In addition, we identified 11 immune cells associated with a decreased risk of TN, including 3 in the Maturation stages of T cell group, 2 in the B cell group, 2 in the TBNK group, 2 in the Treg group, and 1 each in the cDC and Myeloid cell groups. The most significant association was found with CD25hi %T cell (Treg panel) (OR_IVW: 0.893, 95% CI: 0.813 to 0.980, P_IVW = 0.017, P_BW = 0.007) (Fig. 4C).

### Reverse MR analysis

**Fig. 5:**
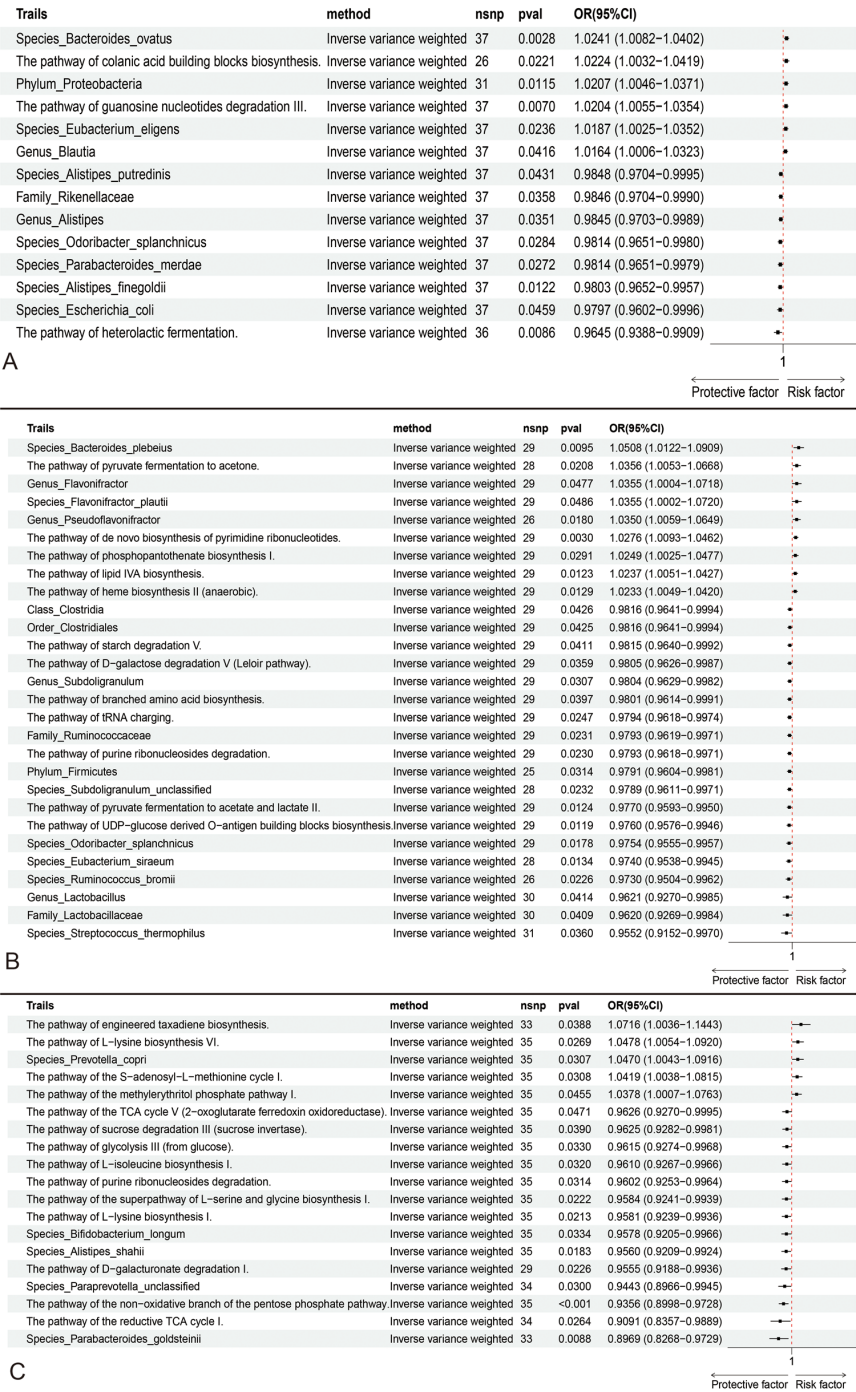
Reverse MR analysis. **A**: MR analysis of PDPN and GM (IVW). **B**: MR analysis of PHN and GM (IVW). **A**: MR analysis of TN and GM (IVW). *CI* confidence interval, *MR* Mendelian randomization, *OR* odds ratio, *nsnp* single nucleotide polymorphism, *IVW* inverse variance-weighted

In reverse MR analysis, we discovered a reverse causal relationship between PHN and 28 types of gut microbiota, with PHN leading to a reduction in the abundance of 19 of these microbes. When TN is considered as the exposure, 19 types of GM are affected in reverse by TN. For PDPN, 14 types of GM are influenced by PDPN. Notably, only two types of GM were significant in both the forward and reverse MR analyses for PDPN: *Proteobacteria* (OR_rIVW: 1.020, 95% CI: 1.005 to 1.037, P_IVW = 0.012) and *Parabacteroides merdae* (OR_rIVW: 0.981, 95% CI: 0.965 to 0.998, P_IVW = 0.027). (Fig. 5).

### Mediation analysis

To ensure the rigor of our findings, we meticulously screened data used in our mediation analyses. For the MR analysis between gut microbiota and study outcomes, we selected data that showed positive associations in at least three statistical methods, including IVW and BWMR, with at least one other method confirming these results. This selection ensured the consistency and reliability of the data, which also had to demonstrate no reverse causality and be free from heterogeneity and pleiotropy issues. Similarly, for the MR analysis between immune cells and study outcomes, we chose immune cells data that showed positive results in both the IVW method and Bayesian weighted analysis, and was free from heterogeneity and pleiotropy, to act as potential mediating variables. This careful selection process guaranteed that the mediation analysis data were consistent, reliable, and representative.

Based on the above data selection process, our analysis results are as follows: we analyzed immune cells as mediators in the pathway from gut microbiota to PDPN, PHN, and TN. Our study indicates that the degradation pathway of fucose mediated by CD4 on CM CD4 + (Maturation stages of T cell) accounts for a 6.7% reduction in PHN risk (total effect beta: −0.966, direct effect GM to immune cells: 0.239, direct effect immune cells to outcome: −0.272, mediation effect: −0.065). CD28 + DN (CD4-CD8-) AC (Treg) associated with *Roseburia inulinivorans* of the *Roseburia* genus accounts for a 12.6% reduction in PHN risk (total effect beta: −1.356, direct effect GM to immune cells: 0.387, direct effect immune cells to outcome: −0.441, mediation effect: −0.171). CD45 on lymphocyte (Myeloid cell) associated with the superpathway of acetyl-CoA biosynthesis increases the risk of TN by 12% (total effect beta: 0.283, direct effect GM to immune cells: 0.249, direct effect immune cells to outcome: 0.135, mediation effect: 0.034). HLA DR + CD8br %T cell (TBNK) associated with the biosynthesis superpathway of GDP-mannose into O-antigen building blocks reduces the risk of TN by 3.3% (total effect beta: −0.366, direct effect GM to immune cells: 0.217, direct effect immune cells to outcome: −0.056, mediation effect: −0.012). IgD-CD38-AC (B cell) associated with thiazole biosynthesis I pathway reduces the risk of PDPN by 7.5% (total effect beta: −0.759, direct effect GM to immune cells: 0.226, direct effect immune cells to outcome: −0.252, mediation effect: −0.057) (Table 1).

**Table 1:** Mediation effect

Mediation effect			
Exposure	Mediator	Outcome	Mediated proportion (%)
Thiazole biosynthesis I pathway	CD45 on IgD-CD38-AC (B cell)	DPNP	7.49
The degradation pathway of fucose	CD4 on CM CD4 + (Maturation stages of T cell)	PHN	6.72
*Roseburia inulinivorans* of the *Roseburia* genus	CD28 + DN (CD4-CD8-) AC (Treg)	PHN	12.61
The superpathway of acetyl-CoA biosynthesis	CD45 on lymphocyte (Myeloid cell)	TN	12.01
He biosynthesis superpathway of GDP-mannose into O-antigen building blocks	HLA DR + CD8br %T cell (TBNK)	TN	3.3

*PDPN* painful diabetic peripheral neuropathy, *PHN* post-herpetic neuralgia, *TN* trigeminal neuralgia

### Sensitive analysis

To deepen our understanding of the potential pleiotropy issues identified in our causal estimations, we conducted several sensitivity analyses. Initially, we applied Cochran’s Q test and MR-Egger regression to explore heterogeneity and pleiotropy among different SNPs regarding their causal relationships. The results revealed that in the MR analysis of the gut microbiota and NP, only the preQ₀ biosynthesis pathway (PWY.6703) exhibited horizontal pleiotropy (P_pleiotropy < 0.05) in the MR analysis involving GM and TN. In the MR analysis of immune cells and NP, horizontal pleiotropy was observed in PHN with IMM CD25 on activated and secreting Treg.

Other results from MR-PRESSO and MR-Egger regression did not indicate horizontal pleiotropy, and Cochran’s Q test also did not show significant heterogeneity. The influence of each SNP on the overall causal estimation was verified through leave-one-out analysis. After removing each SNP, we systematically re-conducted MR analysis on the remaining SNPs. The results demonstrated consistency, with no single SNP significantly violating the overall effect between the GM and NP or between immune cells and NP. Detailed data can be found in the supplementary materials, Tables S10–S12.

## Discussion

Taking advantage of the wealth of public genetic data, our research delved into the causal links among 731 features of immune cells, 412 taxa of GM, and NP. This constitutes the inaugural systematic inquiry utilizing MR analysis to investigate how immune cells play a mediating role in the causal associations between GM (including their metabolic pathways) and three varieties of NP. Unlike earlier studies using 16S rRNA gene sequencing, which poorly identified microbial taxa at finer taxonomic levels, our use of metagenomic sequencing enabled precise species-level identification. This significantly advanced our comprehension of microbiota diversity and its specific roles in the gut ecosystem.

Previous research often neglected potential confounders such as socioeconomic status and dietary habits, and failed to thoroughly explore direct links between specific microbes, immune cell types, and NP. Our study addresses this gap. Our findings reveal causal impacts of 53 GM taxa and pathways out of 207 taxa and 205 pathways reflecting microbial composition and activity, as well as 84 immune phenotypes spanning four immune features (MFI, RC, AC, and MP) on PHN, TN, and PDPN. Moreover, we discovered the mediating effects of five immune cells, enhancing our understanding of the complex interactions among GM, the immune system, and NP.

### Gut microbiota and neuropathy pain

Equilibrium between GM and the host is vital for upholding intestinal barrier integrity, defending against pathogenic intrusions, fostering brain development, and ensuring the normal operation of the immune system. Disruption of this delicate equilibrium can precipitate a range of health complications, including metabolic diseases, cardiovascular diseases, and neurological disorders. A growing body of research suggests that the alteration of diseases such as Alzheimer’s, Parkinson’s, traumatic brain injury, depression, and NP may be linked to gut microbiota dysbiosis (Guo et al. 2019).

The microbiota–gut–brain axis highlights the bidirectional communication among the brain, endocrine system, gut, immune system, and GM, maintaining homeostasis within the organism. GMs are directly associated with pain sensation through peripheral and central systems, affecting the neuroexcitability of primary sensory neurons. They may indirectly regulate inflammatory responses by activating non-neuronal cells, including immune cells (Guo et al. 2019; Min 2023; Lin et al. 2020).

Within peripheral nervous system (PNS), metabolites from GM can directly alter the neuroexcitability of primary sensory neurons in the dorsal root ganglia (DRGs). This modulation is mediated through the activation or sensitization of pain-related receptors or ion channels, such as Toll-like receptors (TLRs), transient receptor potential (TRP) channels, γ-aminobutyric acid (GABA) receptors, and acid-sensing ion channels (Lin et al. 2020). In addition, microbial mediators also indirectly influence the activity of primary sensory neurons within DRGs by prompting immune cells like macrophages to release inflammatory mediators (e.g., TNF-α, IL-1β, IL-6), chemokines (e.g., CCL2, CXCL-1), anti-inflammatory cytokines (e.g., IL-4), or neuropeptides (e.g., endogenous opioids) (Guo et al. 2019).

TLRs serve as crucial transmembrane pattern recognition receptors in the innate immune system, capable of identifying exogenous ligands, PAMPs, and damage-associated molecular pattern molecules (DAMPs) (Min 2023). TLRs are expressed not only on immune system cells like leukocytes but also on nervous system cells including neurons, astrocytes, and microglia. Post-tissue injury, the release of DAMPs recognized by receptors such as TLRs triggers an immune response. PAMPs produced by gut microbiota, such as lipopolysaccharides (LPS), lipoteichoic acid (LTA), peptidoglycan (PGN), and β-glucans, serve pivotally in peripheral sensitization during chronic pain conditions. These molecules, by binding to pattern recognition receptors like TLRs on immune cells and sensory neurons in the DRGs, directly influence neuronal excitability or indirectly trigger inflammatory responses through immune cell activation (Lin et al. 2020; Guo et al. 2019; Ustianowska et al. 2022).

### Gut microbiota, immune system, and pain

When discussing the correlation between GM and pain, the involvement of the immune system emerges as a pivotal factor. Association between immune cells and pain sense, particularly crucial involvement of TLR4 in NP progression, has been extensively studied (Bethea and Fischer 2021; Inoue and Tsuda 2018). In vivo experiments in mouse harboring TLR4 gene mutations demonstrated remarkable reductions in pain hypersensitivity and abnormal behaviors in chemotherapy and nerve injury-induced NP (Su et al. 2022; Bethea and Fischer 2021). This underscores the distinct ability of various TLRs to identify PAMPs, with TLR4 specifically recognizing LPS. This recognition triggers a series of signaling pathways that activate microglia, resulting in the secretion of pro-inflammatory mediators such as CCL2, CXCL-1, and IL-1β, thereby driving the progression of NP.

Within the immune system, T cells, particularly Th2-type T cells, play a pivotal role in regulating chronic neuropathic pain (CNP), characterized by elevated levels of anti-inflammatory cytokines IL-10 and IL-4 (Bethea and Fischer 2021; Uçeyler et al. 2007). While CD4 + Th1 cells are conventionally associated with promoting pain, studies also suggest a potential pain-alleviating role for CD8 + T cells (Krukowski et al. 2016; Liu et al. 2014). Mouse models lacking T cells exhibit prolonged chemotherapy induced pain hypersensitivity, whereas the transfer of CD8 + T cells facilitates pain relief. Regulatory T cells (Tregs), as an important T cell subgroup within pain recovery, function by regulating the actions of innate and adaptive immune cells (Bethea and Fischer 2021; Fischer et al. 2019, 2018).

Studies show significant differences in the diversity and abundance of immune cells and microbial composition between individuals with different pain states, such as visceral pain, chronic pelvic pain, fibromyalgia, and knee pain associated with osteoarthritis, and healthy populations (Gonzalez-Alvarez et al. 2023; Dworsky-Fried et al. 2020; Lin et al. 2020). In the context of complex regional pain syndrome (CRPS), patients’ enduring pain is closely associated with heightened activation of microglia in both the spinal cord and brain, alongside a decline in gut microbiota diversity (Dworsky-Fried et al. 2020). Microglia assume a central role in instigating and perpetuating these adaptive alterations within the central nervous system.

While injured or degenerating sensory neurons incite an inflammatory response to trauma, culminating in NP onset, recent investigations suggest that GM may also contribute to the inflammatory processes linked to chronic pain. Notably, metabolic byproducts of GM, such as SCFAs, exert significant regulatory effects on microglia maturation and function. (Dworsky-Fried et al. 2020; Magni et al. 2023).

### MR analysis of immune cells and gut microbiota in neuropathic pain

In this study, we identified *Rothia mucilaginosa* from the *Rothia* genus as a significant factor increasing the risk of PHN. Commonly found in the human oral cavity and upper respiratory tract as part of normal flora, this bacterium is mostly harmless but can cause infections in immunocompromised individuals (Getzenberg et al. 2021; Daoub et al. 2021). It is associated with a variety of clinical conditions, including periodontal disease, respiratory infections, and even bloodstream infections (Daoub et al. 2021; Getzenberg et al. 2021; Shaeer et al. 2017). In addition, we studied the urate biosynthesis and inosine 5'-phosphate degradation pathway, which converts inosine 5'-monophosphate to uric acid (UA) through a series of enzymatic reactions. Elevated UA levels are linked to gout, cardiovascular risk, hypertension, diabetes, and metabolic syndrome (Zhang et al. 2022; Novak et al. 2007; Heda et al. 2021; Gherghina et al. 2022). Soluble urates promote the production of interleukin-1 beta (IL-1β), triggering acute gouty inflammation mediated by the NLRP3 inflammasome activation, which leads to the release of IL-1β by macrophages and neutrophils, resulting in severe pain and joint swelling (Crișan et al. 2016; Zhang et al. 2022). Furthermore, uric acid-induced oxidative stress, a balance disruption between free radicals and antioxidants, exacerbates inflammation and pain (Gherghina et al. 2022). Interestingly, our findings suggest that this pathway may also increase the risk of PHN, a connection not previously demonstrated between this microbial pathway and neuropathic pain.

In our study, an unclassified strain of the genus *Roseburia* emerged as the factors most markedly associated with an elevated risk of TN. Members of the Lachnospiraceae family, to which *Roseburia* belongs, are typically anaerobic, fermentative, chemoheterotrophic bacteria associated with health due to their role as major producers of SCFAs. SCFAs are crucial for modulating the local microbial environment and interacting with the host’s immune system, playing a key role in regulating intestinal inflammation and immune maturation. Various strains of *Roseburia* have diverse effects on metabolism and inflammation and are linked to conditions such as inflammatory bowel disease, type 2 diabetes, and chronic pain (Dekker Nitert et al. 2020; Kulkarni et al. 2021; Goudman et al. 2024; Machiels et al. 2014; Ruan et al. 2022).

It highlights the risk association between *Bacteroides finegoldii* and PDPN. *B. finegoldii* is a significant intestinal microbe typically found in human feces. While generally symbiotic and beneficial in maintaining a healthy gut microbiota, it can cause infections if it escapes the gut. *B. finegoldii* may play a positive role in managing digestive diseases such as inflammatory bowel disease and Clostridium difficile infections, potentially serving as a probiotic to aid in recovery (Wang et al. 2024b). However, *B. finegoldii* was the only strain identified in our study as potentially increasing the risk of PDPN. Conversely, an increased abundance of *Bifidobacterium longum* was associated with a decreased risk of developing PDPN. As a commensal bacterium in the gut, *Bifidobacterium longum* has demonstrated potential in ameliorating colitis in mouse models. Moreover, it has shown beneficial effects in patients experiencing delayed recovery from hepatocellular carcinoma (HCC) by enhancing liver function and facilitating repair (Yu et al. 2024). Clinical trials have demonstrated that oral administration of a probiotic cocktail containing *B. longum* notably diminishes the proportion of HCC patients experiencing delayed recovery. This intervention also leads to shortened hospital stays and enhances 1-year survival rates (Yu et al. 2024). Moreover, *B. longum* has been shown to lower depression scores in patients with irritable bowel syndrome, enhance quality of life, and reduce reactivity in the limbic system by modulating brain activation patterns (Pinto-Sanchez et al. 2017). In research focused on osteoarthritis (OA), administering *B. longum* orally was found to mitigate pain perception in rats with OA, safeguard cartilage from damage, and diminish the expression of inflammatory cytokines and catabolic markers(Oh et al. 2023).

In reverse MR analysis, PHN, TN, and PDPN are found to decrease the abundance of 19, 14, and 8 types of gut microbiota respectively. Notably, the reductions are primarily in the phyla Bacteroidetes and Firmicutes, which constitute over 90% of the gastrointestinal microbiota. Changes in the ratio of these bacterial groups significantly impact health, influencing conditions like obesity and inflammatory diseases. These bacteria are key in breaking down complex polysaccharides and cellulose, producing SCFAs such as butyrate and acetate, which are crucial energy sources for intestinal cells and support gut health (Jandhyala et al. 2015; Valdes et al. 2018). They also interact with the gut immune system to regulate immune responses and the mucosal barrier, impacting autoimmune and inflammatory responses (Round and Mazmanian 2009). Variations in Bacteroidetes proportions are linked to diseases like obesity and inflammatory bowel disease, while Firmicutes influence the gut’s pH and oxygen levels, affecting the growth of bacterial communities and maintaining microbial balance (Round and Mazmanian 2009; Bäckhed et al. 2004). An imbalance in the Firmicutes/Bacteroidetes ratio is associated with obesity and metabolic syndrome, with higher Firmicutes abundance potentially increasing energy intake efficiency, thus impacting energy balance (Turnbaugh et al. 2006). Despite odds ratios around 0.9, monitoring gut microbiota balance in these patients remains clinically important.

Our research suggests that an elevation in CD4 levels on HLA DR + CD4 + cells within the immune system correlates with a decreased PHN risk. The effector functions of CD4 T cells exhibit a high degree of heterogeneity, primarily exerting their influence through the production of a variety of cytokines and chemokines, as well as the expression of different cell surface proteins on other immune cells, infected cells, or pathogens. CD4 T cells can also exhibit cytotoxic effects, directly eliminating pathogens or infected cells. Given their effector cell diversity, CD4 T cells play a crucial role in controlling infections by a wide range of pathogens, including bacteria, viruses, parasites, and fungi (Tippalagama et al. 2021; Ahmed et al. 2018; Jung et al. 2017; Fiszer et al. 1994).Our research findings are consistent with other literature reports, indicating that CD4 can effectively reduce the occurrence of pain and inflammation (Krukowski et al. 2016; Liu et al. 2014).

CD25hi %T cells are identified as a protective factor against TN. Tregs, typically characterized by CD4 + CD25 + Foxp3 + markers, exert their immunomodulatory effects by directly inhibiting the activation of target cells and secreting anti-inflammatory cytokines such as TGF-β and IL-10. Tregs play pivotal roles in various diseases, including immune tolerance, autoimmune disorders, infectious diseases, organ transplantation, and cancer (Cohen and Boyer 2006). Treg cells’ deficiency can precipitate the development of a spectrum of autoimmune and lymphoproliferative disorders, including autoimmune diabetes. Sustaining FOXP3 expression in CD25-high expressing Tregs is critical for inflammation control. Our study reveals that an augmentation in CD25hi %T cell count correlates with a diminished TN risk.

In our MR analysis of PDPN-related immune cells, we discovered that CD39 on monocyte exerts a significant protective effect against PDPN. Monocytes not only act as effector cells, but also serve as precursors to myeloid dendritic cells and macrophages, with their differentiation rate increasing during periods of inflammation activation (Gu et al. 2023; Boyette et al. 2017). CD39 regulates the conversion process from ATP to adenosine, thereby modulating Treg cells activation and impacting cancer growth (Park et al. 2021). In the context of NP, there is ample evidence indicating the involvement of microglia and blood-derived infiltrating macrophages in pain sensing (Austin and Moalem-Taylor 2010). Particularly in the scenario of PDPN, monocytes and M1 polarized macrophages become the primary producers of TNF-α in the peripheral blood under CNP conditions. Notably, individuals with type 1 diabetes exhibited augmented CD39 expression exclusively in those with PDPN, suggesting the engagement of specific inflammatory cascades (O’Brien et al. 2021). Furthermore, we observed that an elevation in HLA-DR expression on DCs exacerbates the risk of PDPN.

The primary objective of our study is to unravel the causal relationship between GM and NP, shedding light on the intermediary role of immune cells in these intricate connections. Through mediation analysis, we revealed that the pathway of fucose degradation significantly reduces the risk of developing PHN, with 6.7% of this effect mediated by the immune cell CD4 on CM CD4 + . Similarly, the *Roseburia inulinivorans*, belonging to the genus *Roseburia*, also reduces the risk of PHN, with 12.5% of the protective effect mediated by the immune cell CD28 + DN (CD4-CD8-) AC. In TN, we observed that the GDP-mannose derived O-antigen building blocks biosynthesis pathway reduces the risk of TN, with 3.3% of this effect mediated by HLA DR + CD8br %T cell. Conversely, the superpathway of acetyl-CoA biosynthesis increases the risk of TN, with 12% mediated by CD45 on lymphocyte cells. Regarding PDPN, our analysis revealed that the pathway of thiazole biosynthesis I in *E. coli* diminishes PDPN risk, with 7.5% of the effect mediated by IgD-CD38-AC. These findings not only deepen our understanding of the intricate connections between the gut microbiota and neuropathic pain but also underscore the pivotal role of specific immune cells in mediating these connections. They enhance our comprehension of the gut–brain axis interactions and provide valuable insights into potential biomarkers and intervention targets for the development of novel therapeutic approaches, particularly in the realm of NP prevention and treatment.

There are several limitations to this study. First, the analysis relied on data exclusively from European populations, potentially constraining the applicability of the results to other ethnic groups. Second, the GWAS data for NP analyzed had a relatively small number of cases, and larger GWAS datasets are needed for future validation. Third, our study utilized aggregated data rather than individual-level data, restricting our ability to explore subgroup causal relationships, such as gender differences. Fourth, the study results indicated that the genetic predisposition to TN mediated through HLA DR + CD8br %T cells was only 3.3%, a relatively low proportion, suggesting the role of other mediators needs further research to be quantified. Fifth, since data for PDPN were substituted with that of PDPN, the interpretation of results necessitates extra caution. Furthermore, the descriptive nature of the pain data constrained our ability to pinpoint specific pain etiologies, potentially influencing the study outcomes.

Although our study has its limitations, we believe it offers valuable insights into the potential relationships between GM and NP, serving as a foundational piece for future investigations. While generalizing our findings across diverse populations and contexts requires caution, we deem this study significant in unraveling the intricate interplay between GM and NP. Moreover, it provides informative cues for the development of novel therapeutic approaches.

## Conclusion

Our study investigates the causal connections linking the gut microbiota, immune cells, and NP ailments. Employing the BWMR approach, we pinpointed immune cells and gut microbiota exhibiting robust causal associations with NP, while also uncovering the mediating roles of five immune cells in bridging the gap between gut microbiota and NP ailments. These findings provide valuable insights for identifying NP biomarkers and developing therapeutic targets.

## Data Availability

The datasets analyzed during the present study are available as GWAS summary data. The immune cell traits (731 in total) can be accessed at GWAS MRC IEU, with the accession numbers Ebi-a-GCST0001391 to Ebi-a-GCST0002121. The summary statistics of 412 gut microbial features (Study accession numbers: GCST90027446 to GCST90027857) used in this article can be downloaded from the NHGRI-EBI GWAS Catalog. The datasets for painful diabetic polyneuropathy (finn-b-DM_POLYNEURO) can be accessed here. The datasets for post-herpetic neuralgia (finngen_R9_G6_POSTZOST) are available here. The datasets for trigeminal neuralgia (finngen_R10_G6_TRINEU) can be accessed here. These datasets were sourced from the FinnGen consortium’s GWAS summary data, accessible at FinnGen.

## Abbreviations

BWMR: Bayesian weighted Mendelian randomization
CI: Confidence intervals
CNP: Chronic neuropathic pain
CRPS: Complex regional pain syndrome
DAMPs: Damage-associated molecular pattern molecules
DMP: Dutch Microbiome Project
DRGs: Dorsal root ganglia
GM: Gut microbiome
GWAS: Genome-Wide Association Studies
HCC: Hepatocellular carcinoma
IV: Instrumental variables
IVW: Inverse variance-weighted
LD: Linkage disequilibrium
LPS: Lipopolysaccharides
LTA: Lipoteichoic acid
MR: Mendelian randomization
NP: Neuropathic pain
PAMPs: Pathogen-associated molecular patterns
PDPN: Painful diabetic peripheral neuropathy
PGN: Peptidoglycan
PHN: Post-herpetic neuralgia
PNS: Peripheral nervous system
SCFAs: Short-chain fatty acids
SNP: Single nucleotide polymorphism
TLRs: Toll-like receptors
TN: Trigeminal neuralgia
TRP: Transient receptor potential
TSMR: Two-sample Mendelian randomization
UA: Uric acid
